# A Systematic Review of the Impact of Energy Insecurity on Mental Health During the COVID-19 Pandemic

**DOI:** 10.7759/cureus.71370

**Published:** 2024-10-13

**Authors:** Naelijwa J Manongi, Ramkumar Rajapandian, Sajida Moti Wala, Esraa M AlEdani, Essa A Samuel, Khoula Ahmad, Ana P Arcia Franchini

**Affiliations:** 1 Family Medicine, California Institute of Behavioral Neurosciences & Psychology, Fairfield, USA; 2 Trauma and Orthopedics, California Institute of Behavioral Neurosciences & Psychology, Fairfield, USA; 3 Internal Medicine, California Institute of Behavioral Neurosciences & Psychology, Fairfield, USA; 4 Dermatology, California Institute of Behavioral Neurosciences & Psychology, Fairfield, USA; 5 Physical Medicine and Rehabilitation, California Institute of Behavioral Neurosciences & Psychology, Fairfield, USA; 6 Research, California Institute of Behavioral Neurosciences & Psychology, Fairfield, USA

**Keywords:** anxiety, covid-19, depression, energy insecurity, energy poverty

## Abstract

Energy is an increasingly important social and public health concern. It is essential for good health and a prerequisite for basic needs in the household. Public health emergencies like COVID-19 have been known to be associated with mental health crises such as increased levels of loneliness, depression, and anxiety. A systematic search of the PubMed, Semantic Scholar, and ScienceDirect databases was conducted. After duplicate removal, title, abstract, and content screening, retrieval, and quality assessment, 11 studies met the criteria for this systematic review of articles. This study highlighted that the COVID-19 pandemic created a mental health crisis associated with various factors, particularly energy insecurity. The social implications for this study show that it is important for individuals and the public health community to recognize the impact that energy insecurity has on the population more specifically recognizing how energy insecurity affects mental health. Public health measures should focus on getting direct help to households that are struggling to keep the home at a safe temperature and pay their energy bills.

## Introduction and background

Housing is where humans spend the majority of their time outside of their place of employment, it is a crucial determinant of health and has been associated with both physical and mental health [1]. Housing insecurity is defined as “limited or uncertain availability, access, or inability to acquire stable, safe, adequate, and affordable housing and neighborhoods in socially acceptable ways” [2]. The coronavirus pandemic (COVID-19) precipitated unemployment and economic hardships in a significant number of US households, resulting in impacted household expenditures and leading to an increase in housing insecurities [3].

Housing insecurity is not an isolated phenomenon but coexists with other problems, including food insecurity, energy insecurity, and unemployment. Energy is an increasingly important social and public health concern, as it is essential for good health and a prerequisite for basic needs such as cooking, lighting, and heating in the household [4]. Energy insecurity is defined as “the lack of access to adequate, affordable, reliable, acceptable, and clean sources of energy for a healthy and sustainable livelihood” [4]. The COVID-19 pandemic brought about an increase in costs for residential energy needs such as heating and cooling. This increase in costs accounts for a higher percentage of household budgets and represents emerging disparities between richer and poorer households [4].

Why is energy security important? Access to affordable, reliable, sustainable, and modern energy is the focus of sustainable development goal number seven (SDG7). SDG7 is one of the 17 global goals set by the United Nations to be achieved by 2030, with the primary focus being to improve universal access to modern energy services and increase the use of renewable energy sources. SDG7 targets by extension promoting mental and human health, through thermal comfort, and reducing air pollution [5]. Achieving SDG7 is vital for promoting health equity, reducing preventable disease, and ensuring that healthcare systems can function efficiently by addressing the social determinants of health, and contributing to better health outcomes and quality of life [5]. This is especially important for those living in the dense urban centers of both developed and developing countries highlighting the need to ensure access to affordable and reliable energy. Energy insecurity has the power to impact an individual’s psychosocial, economic, nutritional, and physical health. Data shows that most households in the United States are at or near the federal poverty line are significantly burdened by energy costs [4]. Many low-income households struggle with energy insecurity largely driven by economic insecurity caused by the restrictive public health mandates placed to fight the COVID-19 pandemic [6]. Previous research has shown that households that have trouble paying fuel bills and poor thermal comfort are independently associated with higher levels of stress [4]. Additionally, another study found that the inability to keep a household at a comfortable temperature due to energy poverty was associated with higher odds of anxiety and depression [7]. A cross-sectional study done in New York City indicated that twenty-seven percent of participants were energy insecure, and this was associated with an increase in poorer mental health [8].

Rapidly growing research has studied the effects of the COVID-19 pandemic on populations’ mental health. A majority of studies concluded that lockdowns contributed to increased levels of loneliness, depression, and anxiety [9]. Public health emergencies have been known to be associated with mental health crises for example, the severe acute respiratory syndrome (SARS) epidemic in 2003 was associated with a 30% increase in suicide rates and anxiety [10]. Depression rates were reported to have increased 3-fold during the COVID-19 pandemic [10]. Given this concern, the World Health Organization (WHO) raised awareness against COVID-related mental health consequences such as depression, anxiety, and suicidal behavior [6].

Mental health consequences of public health emergencies are not evenly distributed across populations. Being of a minority group with low socioeconomic status is associated with a greater burden of poor mental health [11]. Increasingly, research is pointing to energy insecurity as a public health concern. However, the lack of attention to energy-related insecurity, particularly in the United States during a public health crisis, impels the need to further explore this issue to better understand its implications on mental health, and put forth public health measures that will not increase the burden of disease associated with energy insecurity.

The purpose of this review is to examine the relationship between exposure to energy insecurity and mental health status. The hypothesis is that the odds of poor mental health are strongly associated with being exposed to energy insecurity in the household. This study will answer the following question: what effect does exposure to energy insecurity in the household during the COVID-19 pandemic have on the odds of having poor mental health? This study will contribute to the understanding of how energy insecurity can shape mental health and well-being across various households in the United States.

## Review

Methods

The guidelines outlined by the Preferred Reporting Items for Systematic Reviews and Meta-Analyses (PRISMA) statement were adhered to in the performance of this systematic review. First, a question was developed to give the review a focus. Second, a systematic search of three electronic databases was conducted. We explored the following databases: PubMed, Semantic Scholar, and ScienceDirect. There were four medical search terms: mental health, depression, anxiety, and COVID-19. Additionally, there were three energy-related search terms: energy poverty, energy insecurity, and household energy. A systematic search of the databases was then executed, integrating any of the four medical search terms with any of the three energy-related search terms. The term "any medical search term" signifies OR between each search term. The combined search thus indicated an AND relationship between the two categories of search terms.

In total, 209 records were retrieved from the database search. Those records were subsequently imported into the EndNote (Clarivate, London, UK) citation manager for the purpose of collection, storage, and organization. Within this reference management software, three distinct reference groups (duplicated group, included group, excluded group) were created. Initially, 137 duplicate records were eliminated and categorized into the duplicated group prior to screening, utilizing the functionalities provided by EndNote (Clarivate). Following this, the titles and abstracts of 72 articles were screened to discern those articles relevant to the subject matter of this literature review.

The selection criteria for inclusion of studies in this review required that the articles must have examined the association between energy security and mental health dimensions. Additionally, the articles had to be composed in English, and they must have been published within the preceding five years.

**Figure 1:**
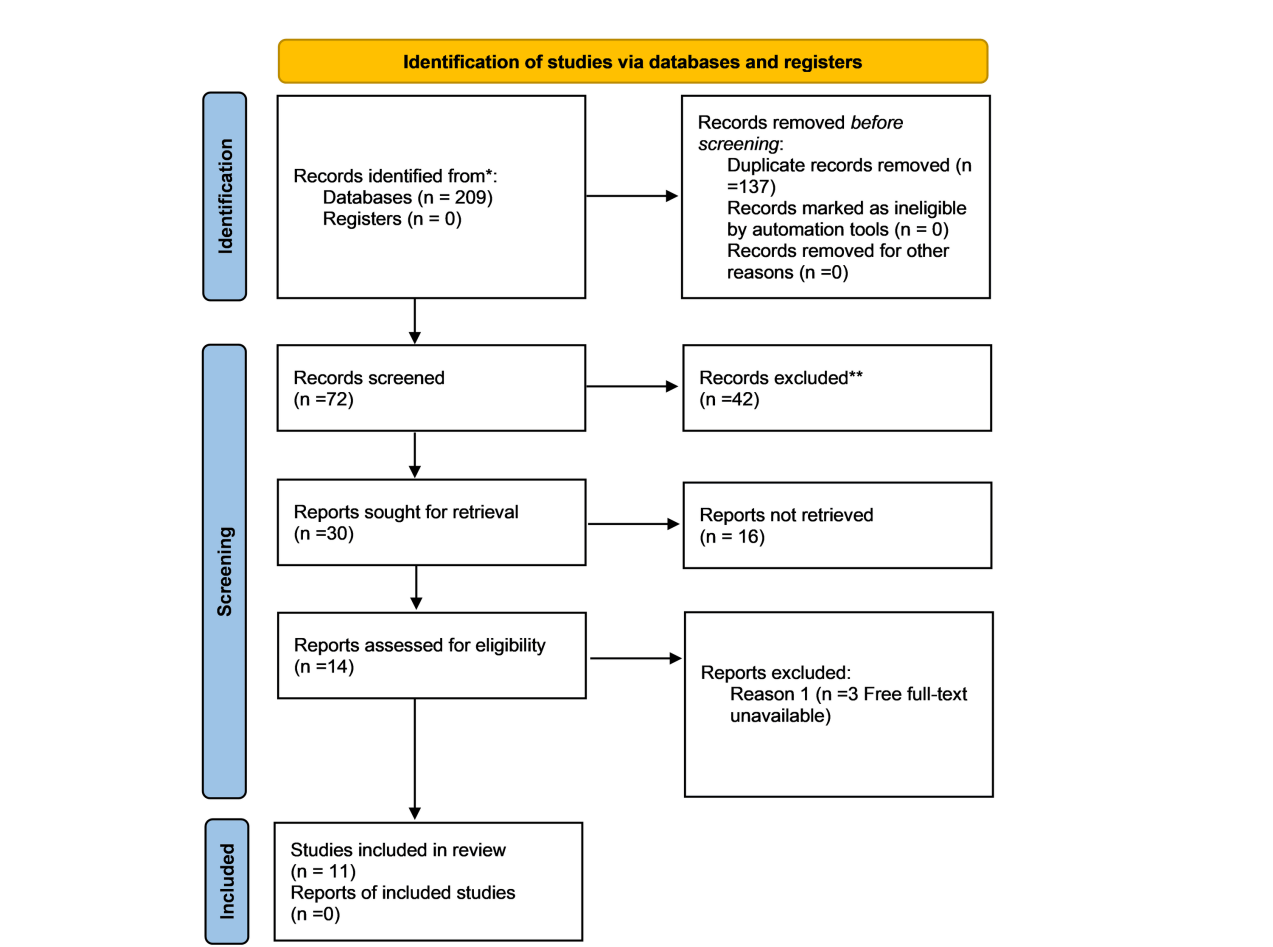
PRISMA 2020 flow chart PRISMA: Preferred Reporting Items for Systematic Reviews and Meta-Analyses

Figure 1 shows a breakdown of the results using a PRISMA flow diagram.

Results

The results of our review were presented according to three key themes identified: (1) exposure to energy insecurity, (2) socio-demographic factors, and (3) mental health status. The presentations of data are organized based on the type of study relevance and future recommendations.

Table 1 includes a snapshot of the 12 studies highlighting the country of findings, themes that were addressed, and relevant results and recommendations that we will discuss further in the discussion section of this systematic review.

**Table 1:** Analysis of selected studies reviewing the impact of energy insecurity on mental health during the COVID-19 pandemic COVID-19: coronavirus pandemic 2019, CCVI: COVID-19 Community Vulnerability index, LEAD: low-income energy affordability data, HILDA: Household Income and Labor Dynamics in Australia, SF-36: short form 36 health survey

Author and year	Focus area	Type of review	Resources included	Themes	Relevant results and recommendations
Mohan, 2021 [7]	Ireland	Non-systematic review	Child and Infant Cohorts of Growing Up in Ireland	Energy poverty, mental health	-Energy poverty increases the likelihood of depression in parents. - Evidence-based policymaking should focus on energy poverty and parental depression.
Xu et al., 2022 [12]	Rural China	Non-systematic review	China Family Panel Studies	Energy poverty, depression	-Energy poverty affects the depression scores of individuals and energy price subsidy may serve as a useful policy instrument.
Boateng et al., 2021 [13]	Global	Non-Systematic review	Sub-Saharan Africa	Energy insecurity, health inequity, COVID-19	-Household energy insecurity is detrimental to the health of the most vulnerable, and the COVID-19 pandemic increased this burden. - Efforts should be targeted to finance and support energy resource security.
Bezgrebeina et al., 2021 [14]	North America	Systematic review	N/A	Housing, climate, health	-Policies targeting structural issues are needed specifically for housing insecurity which will impact population health.
Keller et al., 2022 [9]	Denmark, France, United Kingdom	Meta-analysis	The Danish National Birth Cohort, University College London	COVID-19, housing conditions	-Poor housing conditions are associated with poor mental health.
Batool et al., 2023 [15]	Pakistan	Non -systematic review	Pakistan University students	COVID-19, energy poverty, health poverty	COVID-19 pandemic increased energy poverty in Pakistan. Moreover, energy poverty positively and significantly influences health poverty.
Fefferman et al., 2021 [16]	United States of America	Non-systematic review	U.S COVID Community Vulnerability Index (CCVI), Low-Income Energy Affordability Data (LEAD) Tool, COVID-19 United States cases by county.	Energy policy, COVID-19 pandemic	Low-income households suffered a greater burden of disease; therefore, policies need to address the importance of energy security, and future disaster management.
Mastropietro., 2021 [17]	Spain	Non-systematic review	N/A	COVID-19, energy insecurity, emergency measures	COVID-19 pandemic exacerbated energy poverty. -Emergency measures and economic aid must be flexible and capable of identifying consumers in need of support.
Siegel et al., 2024 [18]	New York City, USA	Non-Systematic review	New York City Household Energy and Health Survey	Energy insecurity, health conditions, mental health conditions	-Unequally socio-demographic distribution of energy insecurity -Using energy insecurity indicators to inform policy.
Lin et al., 2020 [19]	Ghana	Non-systematic review	N/A	Multidimensional energy poverty, mental health, developing country	-Energy poverty heightens the chances of being mentally unhealthy. -Policymakers to adopt a holistic approach in solving issues of energy poverty.
Bentley et al., 2023 [20]	Australia	Non-systematic review	Household, Income and Labor Dynamics in Australia Survey (HILDA)	Energy poverty, mental health	People's mental health substantially worsens (by 4.6 points on the SF-36 mental health measure) when they are unable to pay for home heating.
Riva et al., 2023 [21]	Canada	Non-systematic review	Canadian Housing Survey	Energy poverty, housing, determinants of Health	-Exposure to energy poverty is associated with a significantly increased likelihood of poor general mental health.

Discussion

Energy insecurity is a multifaceted phenomenon that results from a number of variables, including household income and the needs of household members; economic and political factors such as available energy sources, and government policies; and climate and or environmental-related events that can increase or compromise energy needs [21]. Energy insecurity is an overlooked determinant of health.

Over the course of their lives, individuals in poor social situations are more susceptible to poor mental health in ways that are frequently influenced by structural factors that create and maintain intergenerational cycles of poverty and bad health [22]. Previous studies have shown that energy insecurity is highly prevalent among households below the federal poverty line [13]. Addressing these challenges is an imperative matter of social justice and health equity. Energy poverty constitutes a significant public health issue for several reasons. Firstly, it is correlated with both elevated and diminished indoor temperatures which adversely affect health outcomes. Secondly, it is predominantly encountered by the most marginalized communities and households within a societal framework [23].

Measuring Energy Insecurity

Some researchers use various combinations of universally accepted EP indicators, while others try to develop new indices that, in their opinion, better reflect the problem under investigation [24]. The following are the most common questions asked to evaluate energy insecurity. "Does the household keep the home adequately warm?" Two possible options were provided: (i) No, cannot afford; (ii) Yes. The "No, cannot afford" responses were used to construct a variable that represents households that could not afford to adequately heat their homes, categorized as "cold home." Another question asked "Have you ever had to go without heating during the last 12 months through lack of money? (I mean have you had to go without a fire on a cold day, or go to bed to keep warm or light the fire late because of lack of coal/fuel?)" to which there could be either a "yes" or "no" response. "Yes," responses were used to define a variable "gone without heat." The exposure of interest, "energy insecurity," was constructed as a composite from having either a "cold home" and/or "gone without heat."

Measuring Mental Health

To assess mental health, anxiety was measured using the Generalized Anxiety Disorder (GAD)-7 scale, responses to five questions were collected utilizing a Likert scale that spanned from 0-4, subsequently categorized into two distinct values: 0 (not at all) or 1 (a little-extremely) and summed for each subscale. Thus, the summed score for anxiety ranges from 0-4. Severe anxiety was defined as ≥ 3 as suggested by the authors [14].

To measure depression, most researchers used the Patient Health Questionnaire (PHQ)-9 scale for screening, diagnosing, monitoring, and measuring.

The findings show that there was a significant association between energy security and poor mental health status. Our findings indicate that experiencing energy insecurity amid the pandemic is significantly correlated with adverse mental health outcomes, particularly in relation to feelings of anxiety, worry, and depression. The pandemic directly impacted energy insecurity through lockdowns which forced people to stay home, thus increasing household energy consumption and consequently, increasing energy bills, while households were facing a reduction in income, due to unprecedented job loss [19].

Previous research analysis showed evidence of an association between energy insecurity and mental health status throughout the first year of the COVID-19 pandemic while this study was in the year 2023 after public health mandates had been lifted, energy insecurity is still affecting many households [15]. The first year of the pandemic had significant effects on energy security in the United States and worldwide. Lockdowns increased the social determinants of health disparities as people spent more time at home while economic uncertainty made it difficult for individuals to afford energy bills.

Energy poverty has been found to worsen the mental health of the study participants. The significance that clean, reasonably priced, and dependable energy access plays in mental health policy must be considered by policymakers [15]. Energy insecurity is a known risk factor for homelessness, which will further exacerbate poor mental health [21]. In Sub-Saharan Africa, notes Boateng et al., lack of fuel for cooking in homes where the primary food sources are basics that need to be cooked can result in malnutrition, lower calorie intake, and related health issues. Additionally, it can cause the tension, worry, and annoyance that come with energy insecurity to worsen.

Future studies should investigate longitudinal survey designs to measure the persistent nature of energy insecurity and mental health throughout the years from the beginning of the pandemic to current. There should also be a standardized way to measure energy insecurity to yield better results.

Public Health Implications

Public health is a key focus of sustainable development policies in all economies [25]. A healthy society contributes to economic development and greater well-being. The social implications of this study show that it is important for individuals and the public health community to recognize the impact that energy insecurity has on the population and how it affects their mental health. The COVID-19 pandemic created a mental health crisis associated with various factors, particularly energy insecurity. While emergency relief measures exist, such as the Low-Income Home Energy Assistance Program (LIHEAP) [25], they may not have eased the burden on mental health. For example, the LIHEAP program does not give awards directly to households, the grant goes through the state. Targeted households are those with members who are elderly, disabled, or have a young child with this assistance being only once per year, which leaves participating households to devise strategies to budget for higher bills in high-usage months or risk falling behind on their utility bills, repeating the debt cycle [25]. Therefore, there is a need for more energy relief programs and policies.

In the United States, stimulus packages were provided by governments to provide relief to those affected by the pandemic however, this was not enough to mitigate the change of structure of its consumption [26]. Coping strategies need to be employed to aid deficiencies in housing infrastructure, and the inability to consume or afford adequate energy [27]. Many families use various strategies in response to energy insecurity with most forgoing necessities [28]. Goldstein et al., in their paper, recommended on-site clean energy like solar panels in homes as an energy-saving option [29]. Relatively little is known about how energy deprivation affects mental wellness in emerging economies, especially in Sub-Saharan Africa, despite the growing interest in mental health and energy poverty. This is because the majority of these studies have focused on developing economies [21]. In the United States, 31 million households struggle with energy insecurity and burden, and 16 million of these households classify their burden as severe [30]. This shows how important it is for public health measures to focus on getting direct help to households that are struggling to keep the home at a safe temperature and pay their energy bills. Public health mandates placed during a health crisis should not increase health disparities but rather provide equity to all.

Limitations

The available research on the causal relationship between energy poverty and mental health outcomes is limited due to a lack of robust data. Additionally, this systematic review only includes studies published between 2020 and 2024 in the English language; consequently, it may not provide an exhaustive overview of the literature on the topic.

## Conclusions

This literature implies that any effort to tackle the problems of access to and consumption of energy may have a considerable impact on population health. Further studies are needed to study the impact of energy insecurity on mental health status, as well as look across multiple years for trends over time since the start of the COVID-19 pandemic up to now. Local public health communities can address energy insecurity and mental health through intervention programs and policies that assist households that are in need. Policymakers should think about a holistic strategy for addressing energy poverty, wherein all aspects are simultaneously given attention, to minimize energy poverty and enhance mental health.

## References

[1] Marí-Dell'Olmo M, Novoa AM, Camprubí L (2017). Housing policies and health inequalities. Int J Health Serv.

[2] Bhat AC, Almeida DM, Fenelon A, Santos-Lozada AR (2022). A longitudinal analysis of the relationship between housing insecurity and physical health among midlife and aging adults in the United States. SSM Popul Health.

[3] Bushman G, Mehdipanah R (2022). Housing and health inequities during COVID-19: findings from the national Household Pulse Survey. J Epidemiol Community Health.

[4] Hernández D (2016). Understanding 'energy insecurity' and why it matters to health. Soc Sci Med.

[5] Nations U (2024). Goal 7—ensure access to affordable, reliable, sustainable and modern energy for all. https://www.un.org/en/chronicle/article/goal-7-ensure-access-affordable-reliable-sustainable-and-modern-energy-all.

[6] Efstathiou V, Stefanou MI, Siafakas N (2022). Suicidality and COVID-19: suicidal ideation, suicidal behaviors and completed suicides amidst the COVID-19 pandemic (Review). Exp Ther Med.

[7] Mohan G (2022). The impact of household energy poverty on the mental health of parents of young children. J Public Health (Oxf).

[8] Hernández D, Siegel E (2019). Energy insecurity and its ill health effects: a community perspective on the energy-health nexus in New York City. Energy Res Soc Sci.

[9] Keller A, Groot J, Matta J (2022). Housing environment and mental health of Europeans during the COVID-19 pandemic: a cross-country comparison. Sci Rep.

[10] Fang D, Thomsen MR, Nayga RM Jr (2021). The association between food insecurity and mental health during the COVID-19 pandemic. BMC Public Health.

[11] Ettman CK, Abdalla SM, Cohen GH, Sampson L, Vivier PM, Galea S (2020). Prevalence of depression symptoms in US adults before and during the COVID-19 pandemic. JAMA Netw Open.

[12] Xu W, Xie B, Lou B, Wang W, Wang Y (2022). Assessing the effect of energy poverty on the mental and physical health in China—Evidence from China family panel studies. Front Energy Res.

[13] Boateng GO, Phipps LM, Smith LE, Armah FA (2021). Household energy insecurity and COVID-19 have independent and synergistic health effects on vulnerable populations. Front Public Health.

[14] Amerio A, Brambilla A, Morganti A (2020). COVID-19 lockdown: housing built environment's effects on mental health. Int J Environ Res Public Health.

[15] Batool K, Zhao ZY, Sun H, Irfan M (2023). Modeling the impact of energy poverty on income poverty, health poverty, educational poverty, and environmental poverty: a roadmap towards environmental sustainability. Environ Sci Pollut Res Int.

[16] Fefferman N, Chen CF, Bonilla G, Nelson H, Kuo CP (2021). How limitations in energy access, poverty, and socioeconomic disparities compromise health interventions for outbreaks in urban settings. iScience.

[17] Mastropietro P (2022). Energy poverty in pandemic times: fine-tuning emergency measures for better future responses to extreme events in Spain. Energy Res Soc Sci.

[18] Siegel EL, Lane K, Yuan A, Smalls-Mantey LA, Laird J, Olson C, Hernández D (2024). Energy insecurity indicators associated with increased odds of respiratory, mental health, and cardiovascular conditions. Health Aff (Millwood).

[19] Lin B, Okyere MA (2020). Multidimensional energy poverty and mental health: micro-level evidence from Ghana. Int J Environ Res Public Health.

[20] Bentley R, Daniel L, Li Y, Baker E, Li A (2023). The effect of energy poverty on mental health, cardiovascular disease and respiratory health: a longitudinal analysis. Lancet Reg Health West Pac.

[21] Riva M, Kingunza Makasi S, O’Sullivan KC, Das RR, Dufresne P, Kaiser D, Breau S (2023). Energy poverty: an overlooked determinant of health and climate resilience in Canada. Can J Public Health.

[22] Kirkbride JB, Anglin DM, Colman I (2024). The social determinants of mental health and disorder: evidence, prevention and recommendations. World Psychiatry.

[23] Memmott T, Carley S, Graff M, Konisky DM (2021). Sociodemographic disparities in energy insecurity among low-income households before and during the COVID-19 pandemic. Nat Energy.

[24] Bezgrebelna M, McKenzie K, Wells S (2021). Climate change, weather, housing precarity, and homelessness: a systematic review of reviews. Int J Environ Res Public Health.

[25] Bednar DJ, Reames TG (2023). Fleeting energy protections: state and utility level policy responses to energy poverty in the United States during COVID-19. Energy Res Soc Sci.

[26] Shao Q (2023). Pathway through which COVID-19 exacerbates energy poverty and proposed relief measures. Energy Sustain Dev.

[27] Graff M (2024). Addressing energy insecurity: policy considerations for enhancing energy assistance programs. Heliyon.

[28] Hernández D, Laird J (2022). Surviving a shut-off: U.S. households at greatest risk of utility disconnections and how they cope. Am Behav Sci.

[29] Goldstein B, Reames TG, Newell JP (2022). Racial inequity in household energy efficiency and carbon emissions in the United States: An emissions paradox. Energy Res Soc Sci.

[30] Baker SH, Carley S, Konisky DM (2021). Energy insecurity and the urgent need for utility disconnection protections. Energy Policy.

